# Restricted thumb extension due to flexor pollicis longus tendon adhesion following volar plate fixation with bone substitute use for distal radius fracture: A case report and literature review

**DOI:** 10.1097/MD.0000000000045727

**Published:** 2025-10-31

**Authors:** Youn-Tae Roh, Chia Jan Wang, Il-Jung Park

**Affiliations:** a Department of Orthopaedic Surgery, H Plus Yangji Hospital, Seoul, Republic of Korea; b Department of Orthopaedic Surgery, Bucheon St. Mary’s Hospital, College of Medicine, The Catholic University of Korea, Seoul, Republic of Korea.

**Keywords:** bone graft substitutes, distal radius fracture, flexor pollicis longus tendon, tendon adhesion

## Abstract

**Rationale:**

Tendon adhesion is a relatively uncommon complication after distal radius fracture surgery.

**Patient concerns:**

A 51-year-old woman underwent open reduction and volar plate fixation for a distal radius fracture at another hospital approximately 4 weeks ago. Upon presentation to our hospital, active flexion of the thumb interphalangeal (IP) joint was preserved, but active extension was absent; additionally, passive extension of the thumb IP joint could not be achieved by the examiner.

**Diagnoses:**

Plain radiography and computed tomography demonstrated that 1 distal screw of the volar plate protruded beyond the dorsal cortex, with radiopaque material visible on the radial-volar aspect of the plate. Although rupture of the extensor pollicis longus tendon by the protruding screw was considered, passive extension of the thumb IP joint is generally preserved in such cases. Therefore, adhesion of the flexor pollicis longus tendon was considered more likely than extensor pollicis longus tendon rupture.

**Interventions:**

Surgical exploration confirmed severe adhesion of the flexor pollicis longus to the volar plate, which was released via adhesiolysis. And remnants of the demineralized bone matrix and hydroxyapatite complex previously applied were identified and removed.

**Outcomes:**

Early initiation of both active and passive rehabilitation was implemented postoperatively without immobilization. At 4 years postoperatively, the patient demonstrated full active extension of the thumb IP joint, with no evidence of recurrence.

**Lessons:**

When using bone graft substitutes containing demineralized bone matrix or bone morphogenetic proteins, measures should be taken to prevent leakage into adjacent soft tissues, particularly in regions containing multiple tendons. In addition, any leaked bone graft substitute should be removed using techniques such as irrigation at the surgical site.

## 1. Introduction

When thumb extension is absent following volar plate fixation for a distal radius fracture, rupture of the extensor pollicis longus (EPL) tendon is usually suspected, most often due to attritional injury from a dorsally protruding screw or direct tendon injury during drilling.^[1–4]^ Adhesion of the flexor pollicis longus (FPL) tendon may also cause loss of thumb extension, typically resulting from excessive surgical manipulation or restricted early motion due to prolonged immobilization, although this condition is rarely encountered in routine clinical practice.

Here, we report a case of significant FPL tendon adhesion following volar plate fixation for a distal radius fracture performed at another institution, which led to restricted thumb extension. During revision surgery, severe FPL adhesion and persistent remnants of demineralized bone matrix (DBM) and hydroxyapatite (HA) anterior to the volar plate were identified and removed. Although it is difficult to determine the exact cause of FPL tendon adhesion, we propose that these bone substitute materials around the tendons may have contributed to the development of adhesion. To the best of our knowledge, this is a very rare lesion that has not been previously reported. Written informed consents were obtained from the patient for publication of this case report and the accompanying images.

## 2. Case presentation

**Figure 1.**
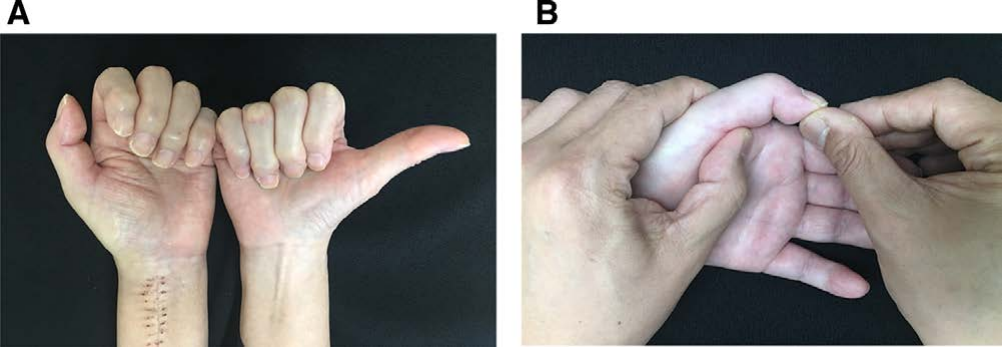
Clinical photographs reveal that (A) active extension of the left thumb interphalangeal joint could not be performed, and (B) passive extension of the left thumb interphalangeal joint by the examiner was also not possible.

**Figure 2.**
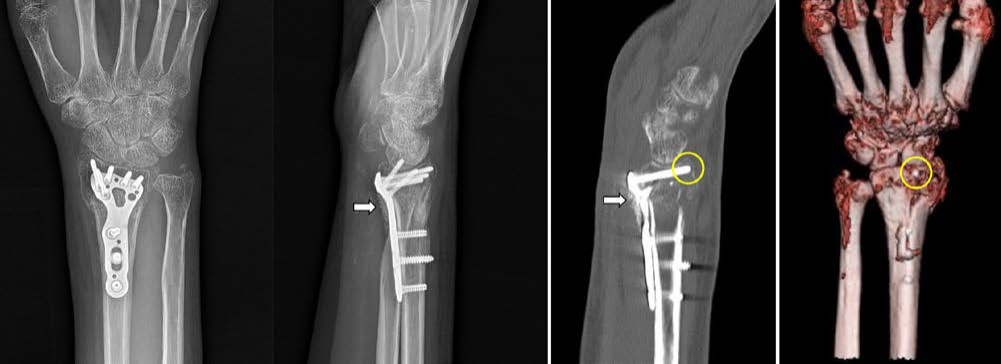
Plain radiographs and computed tomography demonstrated that one of the distal screws of the volar plate was protruding beyond the dorsal cortex (yellow circle), and radiopaque material was visible along the volar-radial aspect of the plate (white arrow).

A 51-year-old female patient underwent open reduction and internal fixation with volar plate for a distal radius fracture at another hospital approximately 4 weeks ago. Approximately 2 weeks postoperatively, it was noted that the patient was unable to extend her thumb. The patient reported no other medical conditions, including diabetes, corticosteroid use, smoking, or alcoholism. Upon presentation to our hospital, physical examination revealed preserved active flexion of the thumb interphalangeal (IP) joint, but active extension was absent (Fig. 1A). In addition, passive extension of the thumb IP joint could not be achieved by the examiner (Fig. 1B). Plain radiography and computed tomography performed at our facility demonstrated that 1 distal screw of the volar plate protruded beyond the dorsal cortex, with radiopaque material visible on the radial-volar aspect of the plate (Fig. 2). Magnetic resonance imaging (MRI) or ultrasound is often useful for evaluating soft-tissue pathology. However, MRI was not feasible because the internal fixation device can cause MRI artifacts (metal-induced artifacts in MRI). Such artifacts can obscure anatomic structures, appear as bright or dark signal changes, or cause geometric distortion due to magnetic field interactions and material properties. Although rupture of the EPL tendon due to the protruding screw was considered, passive extension of the thumb IP joint is typically maintained in such cases. Based on these findings, adhesion of the FPL tendon was considered more likely than EPL tendon rupture. A review of the medical records from the previous hospital confirmed that Penoss® (Hansbiomed, Seoul, Republic of Korea), a composite material consisting of DBM, allograft bone, and HA, was crushed and utilized to fill the bone defect area during surgery.

**Figure 3.**
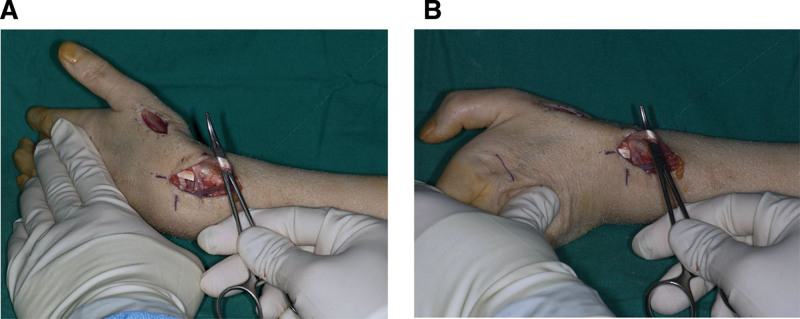
The intraoperative photographs illustrate that (A) with the wrist in flexion, complete extension of the thumb interphalangeal joint was attained following pulling of the extensor pollicis longus tendon, while (B) with the wrist in extension, pulling of the extensor pollicis longus tendon failed to elicit extension of the thumb interphalangeal joint.

**Figure 4.**
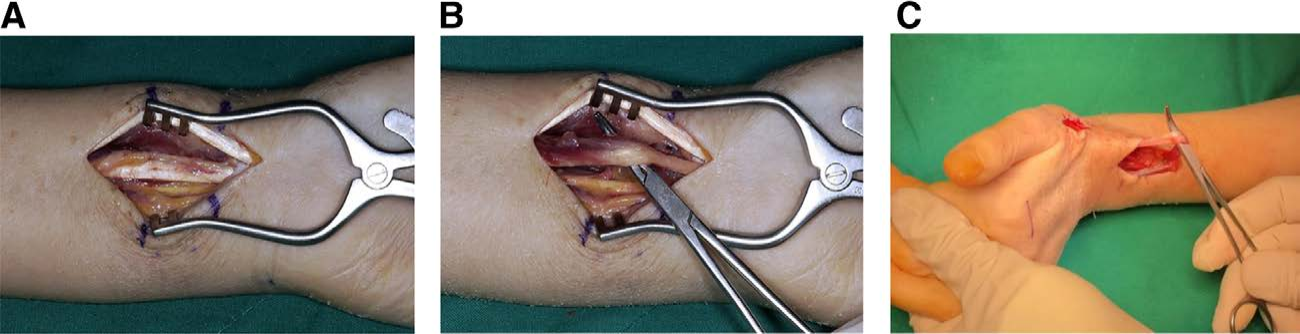
Intraoperative images reveal that (A) marked adhesions were present around the flexor pollicis longus tendon, (B) adhesiolysis was subsequently performed. (C) With the wrist positioned in extension, full extension of the thumb interphalangeal joint occurred upon pulling of the extensor pollicis longus tendon.

Due to concerns regarding adhesion of the FPL tendon and in order to rule out rupture of the EPL tendon, surgical intervention was undertaken. Under axillary nerve block anesthesia, a longitudinal incision was made over the dorsal aspect of the wrist at the level of Lister tubercle to expose the EPL tendon. Inspection revealed that the EPL tendon was intact, showing no rupture and maintaining good continuity. With the wrist flexed, passive manipulation of the EPL tendon produced full extension of the thumb IP joint (Fig. 3A). In contrast, with the wrist extended, passive traction of the EPL tendon did not produce thumb IP extension (Fig. 3B), suggesting flexor tendon adhesion. Accordingly, a volar exploration was performed through the prior volar incision. The FPL tendon had mild adhesions to adjacent neurovascular structures (e.g., median nerve and radial artery) but was firmly adherent to the volar plate, restricting gliding motion. The tendon was released from the surrounding tissues and the plate. Scarring occurred where the tendon contacted the volar plate, requiring debridement (Fig. 4A and B). Remnants of the previously applied DBM/HA composite were identified between the FPL tendon and the volar plate and were completely removed. Following this procedure, passive traction of the EPL tendon with the wrist extended enabled full extension of the thumb IP joint (Fig. 4C).

**Figure 5.**
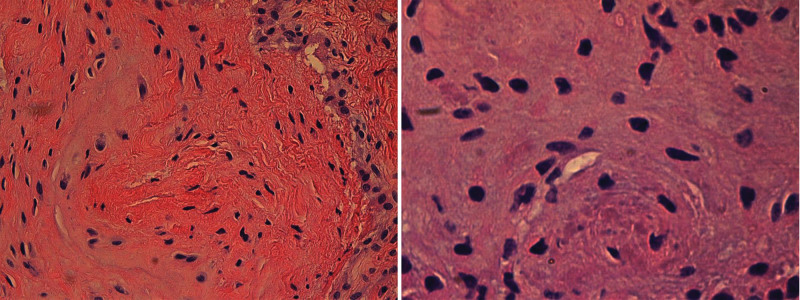
Photomicrography reveals fibrosis with myxohyaline degeneration within the adhesion tissue (H&E staining, x40, x100).

**Figure 6.**
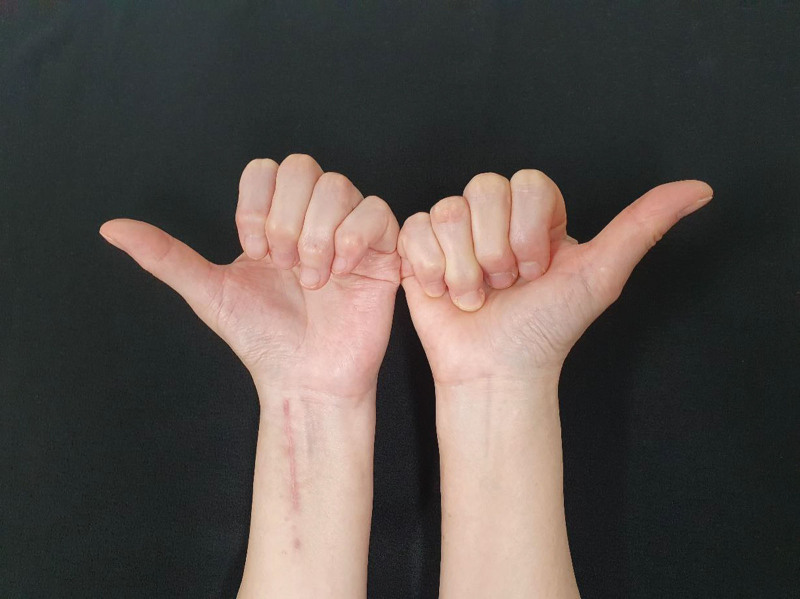
The clinical photograph demonstrates that complete active extension of the thumb interphalangeal joint was preserved at postoperative follow-up.

A volar cock-up splint was applied for 3 days after surgery. The patient was then encouraged to begin active hand and wrist range-of-motion exercises. Instead of engaging a hand therapist, an orthopedic surgeon provided education on self-directed home exercise programs and closely monitored progress during clinic visits. Full activity was permitted approximately 1 month post-surgery. Histopathological analysis of the adhesion tissue demonstrated fibrosis with myxohyaline degeneration (Fig. 5). Four years after surgery, the patient maintained full active extension of the thumb IP joint, with no evidence of recurrence (Fig. 6). The visual analogic scale pain score was 1.0 and pinch strength was 90% of the contralateral side. The disabilities of the arm, shoulder and hand (DASH) score was 4.5 and Michigan hand outcomes questionnaire (MHQ) score was 92.

## 3. Discussion

Distal radius fracture is the most common fracture of the upper limb. With advances in surgical techniques and implants, operative management has become increasingly favored. Over the past 2 decades, treatment has shifted from percutaneous pinning or external fixation to open reduction and internal fixation using a volar locking plate. However, the complication rate associated with volar locking plate fixation is estimated at approximately 30.8%, with tendon rupture representing the most significant tendon-related complication.^[5]^ When thumb extension is compromised after distal radius fracture surgery, EPL tendon rupture is the most common cause. Nevertheless, as demonstrated in this case, FPL tendon adhesion may also contribute to this condition. Comprehensive physical examination is essential to distinguish between these pathologies. In EPL rupture, patients lose active thumb extension while passive extension remains preserved, resulting in an extension lag at the IP joint. In contrast, FPL adhesion manifests as a flexion contracture of the thumb IP joint, limiting both active and passive extension. A noteworthy clinical feature of FPL adhesion is that passive extension of the thumb IP joint can be achieved when the wrist is flexed, but not with the wrist extended.

Rupture of the EPL tendon following volar plate fixation for distal radius fractures is relatively common, with proposed mechanisms including attritional injury due to a dorsally protruding screw or direct damage resulting from drilling.^[1–4]^ In contrast, reports detailing FPL adhesion are relatively limited. Chiu et al described a case of FPL tendon entrapment by a plate during minimally invasive plate osteosynthesis.^[6]^ Thione et al reported an instance of FPL entrapment following conservative management of a distal radius fracture, attributing the pathology to fibrosis caused by bleeding at the fracture site or direct entrapment of muscle tissue.^[7]^ Geissler et al presented a pediatric forearm fracture with mechanical entrapment of both the FPL and the flexor tendon of the index finger, resulting in flexion contractures mimicking Volkmann ischemic contracture.^[8]^ Each of these complications may result in restricted thumb extension after distal radius fracture, but they are distinct from the FPL adhesion observed in our patient. Previous literature suggests that tendon adhesion may result from excessive surgical manipulation or restricted early joint movement due to prolonged immobilization.^[9]^ In our case, however, the patient began active finger motion immediately after the initial surgery.

Autologous bone grafting remains the gold standard for addressing bone defects in fracture management; however, multiple bone graft substitutes have gained widespread use. DBM, valued for its osteoinductive properties, contains multiple bone morphogenetic proteins (BMPs).^[10]^ In this case, DBM, allograft, and an HA composite in the form of PenOss® were applied during the initial surgery and were later observed along the voloradial aspect of the plate. PenOss is a cylindrical stick–type composite (3.5 mm in diameter and 20 mm in length) typically used in spine surgery to enhance pedicle screw fixation, but in extremity fractures it can be crushed and packed into bone defects similar to allogeneic bone chips. The operative records did not document the exact method of application, so the precise volume and placement remain unknown. For distal radius fractures, PenOss is generally inserted within the bone through the voloradial cortex fracture gap. Unlike allogeneic bone chips, which are usually packed only until the defect is filled, PenOss contains DBM and may therefore be implanted in relatively larger amounts, allowing material to escape into extraosseous soft tissue or leaving some intentionally outside the bone. Although it is difficult to determine the exact cause of FPL tendon adhesion, we propose that these bone substitute materials around the tendons may have contributed to the development of adhesion. Some of the graft materials may have escaped from the bone defect site and come into direct contact with the FPL tendon, potentially causing adhesion through a fibrotic response or heterotopic ossification mediated by growth factors such as transforming growth factor-β1 (TGF-β1) or BMPs. TGF-β1 has a well-established role in tendon adhesion, and numerous in vivo and in vitro studies have demonstrated that it can induce excessive scar formation leading to adhesion,^[11]^ while inhibition of TGF-β1 has been shown to effectively reduce adhesion.^[12,13]^ Furthermore, the role of BMPs in tendon adhesion remains to be fully elucidated,^[11]^ but current studies have reported that exogenous delivery of BMPs can promote bone ingrowth at the tendon-to-bone junction,^[14]^ and elevated levels of BMPs have also been shown to induce heterotopic ossification.^[15]^ Although the direct involvement of DBM components in the early stages of tendon adhesion is not fully understood, this potential mechanism should be investigated further. Bone graft substitutes containing DBM or BMPs are effective for reconstructing bone defects; however, clinicians should be aware of the risk of heterotopic ossification or tendon adhesion when employing these materials. Accordingly, meticulous attention must be paid to avoid contact of these bone graft substitutes with adjacent soft tissues.

## 4. Conclusions

Although uncommon, FPL tendon adhesion should be recognized as a potential complication after distal radius fracture surgery. When using bone graft substitutes containing DBM or BMPs, meticulous technique is required to prevent leakage into adjacent soft tissues, particularly in regions with multiple tendons. And it is important to remove any leaked bone graft substitutes using techniques such as irrigation at the surgical site.

## Acknowledgments

We thank Chang Deok Weon, a medical photographer at Bucheon St. Mary hospital, the Catholic University of Korea for help in preparing the photographs.

## Author contributions

**Conceptualization:** Youn-Tae Roh, Il-Jung Park.

**Data curation:** Il-Jung Park.

**Funding acquisition:** Chia Jan Wang, Il-Jung Park.

**Methodology:** Youn-Tae Roh, Il-Jung Park.

**Software:** Youn-Tae Roh, Il-Jung Park.

**Supervision:** Il-Jung Park.

**Validation:** Youn-Tae Roh.

**Writing – original draft:** Youn-Tae Roh, Chia Jan Wang.

**Writing – review & editing:** Il-Jung Park.
